# Bibliographical Notices

**Published:** 1838-04-01

**Authors:** 


### Periscope;

OR,

### CIRCUMSPECTIVE REVIEW.

"Ore trahit quodcunque potest, atque addit acervo."

### Notices of some New Works.

TRAITÉ THEORIQUE ET PRATIQUE DE LA DERIVATION, &c.--A THEORETICAL AND PRACTICAL TREATISE ON DERIVATION IN AFFECTIONS THE MOST COMMON, AS PLETHORA, INFLAMMATION, HÆMORRHAGE, &c. By *L. F. Gondret*, M.D. 8vo. pp. 331. *Paris*, 1837.

IT would not require a great deal of ingenuity to render it highly probable that nine-tenths of our most scientific, as well as empirical *medication*, consist of derivation, counter-irritation, or revulsion. Every remedy, or nearly so, applied to the external or internal surface of the body, acts in one or other of these ways. We might perhaps add some of the deadly sedatives, as prussic acid, belladonna, or the poison of some animals—whose operations are inscrutable. But these form a small part of our magazine of remedial agents. Even specifics, as mercury, operate, in all probability, in the way above-mentioned. Mercury irritates or stimulates the liver and numerous other glands, by which a revulsion is made from diseased or disordered structures, and relief obtained. Jalap, sulphur, and all purgatives, effect counter-irritation in the line of the alimentary canal, and consequently revulsion from numerous other parts of the living machine. Blisters, rubefacients, and all external applications of that description, are acknowledged to be medicinal entirely from the operation in question. St. John Long *derived* his income, fame, and even his sepulchre, from counter-irritation—and Baron Dupotet is now gathering his golden opinions, if not guineas, by revulsion of human reason, and excitement of the credulity in both sexes. The author of the little work before us is not undistinguished in his own country as a man of some talent; but like all men of genius, he has a small crotchet in his brain—which is, that the great majority of diseases arise from, or are the products of (*sont produites par*) plethora, inflammation, or hæmorrhage—all of which, he maintains, are but modifications of one morbid process. To these three modifications, however, he adds a fourth, which he considers as something special or specific—a sense of *weight* (*pésanteur*), which he attributes to an overplus of fluid in the part affected, not less indicated by sure signs than the celebrated *rubor*, *dolor*, *calor*, *humor* of the schools. It is always, in his estimation connected with one of the three morbid conditions above mentioned, plethora, &c. He guards against attributing all (only the majority) of maladies to the foregoing states. Genuine typhus (which he has studied since the year 1794) he regards as a cerebral affection, analogous to plethora. By long experience he has become confirmed in this opinion, by the benefit which he saw result from cupping the head, especially along the course of the lambdoidal suture—and dry cupping the lower extremities. He relates cases drawn from the Hôtel Dieu practice so late as 1831.

Dr. G. contemplates "DERIVATION" under two points of view—as normal and therapeutic. The former consists of regular action of all the secretions and excretions—in other words it is sound health. Therapeutic derivation consists in restoring normal derivation, when deranged, by artificial means—such as sudorifics, diuretics, purgatives, &c. From anatomy our author draws the conclusion that the head is supplied with a most extraordinary quantity of blood, in proportion to other parts of the body—the carotids and vertebrals being larger than the two great arteries, carrying blood to the lower extremities. The Doctor protests against the application of leeches and cupping-glasses to the head, in acute diseases of the brain, as calculated to increase rather than diminish the local plethora. However specious may be the theoretical reasonings on this point, experience dispels them at once, because we actually see acute cerebral affections relieved and cured by local depletion in the immediate vicinity of the organ. Dr. G. indeed may say—try the bleeding about the legs and feet when you have phrenitis or apoplexy—and you will find the latter plan the best. Why, it is tried every day in France and Germany;—but we do not think with the success attendant on the English practice. He does not object, however, to cupping-glasses to the nucha, but to leeches on the temples or behind the ears he has a decided antipathy. He thinks the bites of the leeches cause such irritation or inflammation as to attract blood to the head. In chronic diseases of the brain or eyes, Dr. G. objects not to the leeches. He does not apply his reasonings in the same manner to other parts of the body.

On the contrary, when the lower regions of the trunk or members are the seat of plethora, inflammation, hæmorrhage, &c., as in gastro-enteritis, hæmorrhoids, uterine discharges, &c. the surest means of counteracting these affections are, by cupping the back, loins, and shoulders, by way of derivation. Our author, however, trusts greatly to a counter-irritant, called the "POMMADE AMMONIACAL,"—a combination of oil of almonds with caustic ammonia and lard, in the following proportions:—

R. Axungiæ . . . . . . . . ℥vii.

Ol. Amygdal . . . . . . . ℥iss.

Liquor. Ammon. . . . . . . ℥v. vel. ℥vj.

Misce ft. Linimentum.

Upon this counter-irritant, the Academy of Sciences charged MM. Portal, Thenard, Percy, and others, to make a report, which is given in this volume. They observe that this POMMADE may be irritant, rubefacient, vesicatory, or escharotic, according to the mode of application, or the indications for which it is employed. These effects, they observe, are regular, constant, and easily obtained. They consider the remedy as very useful, and applicable to a great many diseases. Absorption never takes place apparently, and the utility depends on the counter-irritation. In this respect they estimate it as much superior to the Cantharides.

We need not follow our Author through the long catalogue of acute and chronic maladies in which he has employed the "POMMADE"—the actual cautery—and the removal of atmospheric pressure by cupping-glasses. There can be no doubt that this class of remedies has been too much neglected in this country—partly from laziness to superintend an external application,—but principally from the unhappy mode of remuneration for polypharmacy instead of advice. We do not suppose that the POMMADE is superior, or even equal, to the antimonial ointment; but the practice of counter-irritation and derivation ought to be encouraged, because it is not sufficiently extended in these Isles. It is, however, increasing; and we believe that the charlatannerie of St. John Long has been beneficial in this respect, as stimulating British practitioners to employ means which were very successful in the hands of a quack.

But the removal of atmospheric pressure, by means of the ingenious contrivance exhibited by Sir James Murray last year, in Liverpool, is what we are anxious to draw attention to at this time. The dry-cupping is a poor and a painful substitute for the pump-apparatus of Sir James. This powerful machine is capable of producing the most extraordinary derivation of blood from one part of the body to the other. Almost all the fluids of the head and trunk might be drawn to the lower extremities by this apparatus, and retained there as long as the operator chose. We have little doubt that, ere many years, this apparatus will be in the hands of most medical practitioners.

---

Arsenicated Candles. Extracts from the Report of the Westminster Medical Society.

The ingenuity, not to say the wickedness of man, in devising means of fraud and adulteration in this enlightened age, is not very creditable to the human heart, however complimentary it may be to the head. The Arsenicated Candle is one of the latest poisons thrown into the cauldron for the *consumption* of the community; and much credit is due to the Westminster Medical Society, and especially to Mr. Everitt, for detecting the enemy, and throwing a *light* on this dangerous adulteration. To Dr. Scott, of the Strand, the Society and the community are also under considerable obligations for the trouble and expense attendant on the experiments at his house. But the public are more indebted to Dr. Granville, than to any other individual, either of the Society, or the Committee, for the admirable Report which he has drawn up, almost entirely from his own researches, observations, and reflections. We regret that our space will not permit us to give the whole document; but our extracts will be sufficient to convey much of the spirit as well as the material of the paper.

"Those who have followed the progress of analytical and organic chemistry for the last quarter of a century, need not be reminded, that Chevreul, one of the most distinguished French chemists of the present day,—in the course of a laborious and interesting investigation into the nature and elementary composition of animal fats—an investigation which lasted ten years, and has been characterised by Berzelius as the most complete and most perfect that has ever been undertaken by chemists—discovered that common tallow consisted of two distinct substances: the one solid, the other of the consistence and appearance of olive oil; to the former of which he assigned the name of *Stearine*, (tallow) and to the second that of *Elaine*, (oil.)

The first of these substances, the only one with which the Committee had any concern, when modified by saponification, crystallizes (in its pure state) in long brilliant and silky needles, arranged in large compact masses, beautifully white, almost odourless, and resembling, in a great degree, those square lumps of pure spermaceti, which may be seen exhibited in the windows of wax-chandlers' shops in London. With these physical advantages over ordinary tallow, and another also, namely, that of resisting a much higher temperature without melting, it might have been expected that this modified Stearine, as it will be called for brevity's sake in the course of this Report, or solid Stearic acid, would soon have been employed for the purpose of fabricating a superior sort of candles. Such, however, was not the case, until several years after its discovery, namely, about six or seven years ago, when Stearine candles were first manufactured in Paris; for they appeared to have formed part of the exhibition of the products of French industry, which took place in that capital in 1834, and which has been described in a work, in three vols. 8vo., by Baron Charles Dupin.

From authentic documents in their possession, your Committee learn, that the mode of manufacturing candles with Stearine, had at first presented difficulties, which were only overcome by some process that was kept a profound secret in Paris; and a knowledge of which was sold some time after to a London manufacturer, who immediately introduced it into this country. The names of the parties are known to your Committee, and will be found in the minutes of their meetings, as well as among the documents laid before the Society; but the introduction into this Report of such names, or that of any other manufacturers of Stearine candles, under whatever denomination they be sold, has been deemed unnecessary and unadvisable.

Although Stearine was prepared in London in considerable quantity, candles manufactured with it were not, at first, so numerous, as they have become since; owing to the secret process employed, to render them fit for sale. Your Committee have been informed, by a very intelligent manufacturer of candles, who made use of, but has since given up the process in question, that an individual in possession of the secret, went about to the candle-makers in London, to sell, for a consideration, not only the mode, but the very material, with which Stearine was to be made fit to be converted into showy and attractive candles for the market.

The material in question was very soon ascertained by some of the candle-makers to be powdered white arsenic; and thus having emancipated themselves from the tax, which they formerly paid for what had now ceased to be a secret process,—those persons were enabled to extend the field of their operations, whereby the practice of manufacturing Stearine candles with arsenic, became very soon almost general. Nay, such was the effect produced on the market by the appearance of this novel and extensive branch of trade, (at a price lower than that of any other candle, except those of common tallow,) that some of the most respectable manufacturers of wax and spermaceti candles were compelled to resort to the making of the new Stearine candles, (according to the then well-known process, which your Committee have learned, consisted of putting one pound of white arsenic in every hundred weight of Stearine,) in order to retain their customers, and, in some measure, indemnify themselves for the losses sustained, in consequence of the great diminution that had necessarily followed in the sale of every superior sort of candles.

As is generally the case in all matters of this kind, notwithstanding the notoriety of the practice among the trade, the public, whose welfare was likely to be affected by it, remained in ignorance of the fact, that in using the new Stearine candles they were burning arsenicated candles; until Mr. Everitt mentioned the subject in a lecture delivered in June last, before the Medico-Botanical Society,—and again until Dr. Scott, in October, as stated in the introduction to this Report, brought it to the notice of the Westminster Medical Society, and led them to the present investigation.

The admission made by the parties themselves, who employed it, that arsenic was contained in the candles in question, might have been deemed sufficient for the purpose of the present investigation: but your Committee could not rest satisfied without verifying the fact by chemical analysis, and still less without ascertaining the quantity present in each candle: as that point was held to be of great importance in determining the probable injurious effects of such candles on the human constitution.

Accordingly a great many specimens of the candles in question, were procured from several shops, under various denominations, and were submitted to accurate analysis. This was confided principally to Mr. Everitt, who repeated his experiments before several members of the Committee, and whose results were afterwards corroborated by some fresh experiments made by Mr. Golding Bird, supported by the testimony of Mr. Richard Phillips.

The Society, through the kindness of the first of these three chemists, had an opportunity of witnessing, at one of their ordinary meetings, the repetition of some of the experiments in question; which consisted not only in testing the water (with which the suspected Stearine had been boiled for some time) by proper re-agents denoting the presence of the white oxide of arsenic; but also in reproducing the metallic arsenic from the precipitate that had been obtained in the liquid by means of sulphuretted hydrogen gas.

Through the various experiments which he made, and often repeated, Mr.

Everitt satisfied your Committee that the quantity of white oxide of arsenic, or arsenious acid, contained in the candles submitted to analysis, varied in different samples from ten to eighteen grains in the pound of four candles, and that the largest proportion of it, namely, four grains and a-half in each candle, was found in the specimen which bore the lowest price of sale.

By another set of very ingenious experiments, conducted with the greatest precision, it was ascertained that this quantity of arsenious acid is only mechanically mixed with the Stearine, and not dissolved in it, (the saponified Stearine appearing to be scarcely capable of holding any portion of it in solution:) and it is worthy of remark, that a larger quantity was found at the top of some of the candles, which in the act of moulding forms the lower end in the mould, than at the bottom. The difference between the two ends amounted to nearly one-third of the whole: so that when such a candle is first lighted it must emit a larger quantity of arsenious acid, that when it is nearly burnt out. These several quantities of the poisonous substance are given out during the combustion of the candle, in the form of subtle vapours of arsenious acid, a fact which was proved by the deposits obtained on the inner surface of glass vessels placed over the lighted candle, and which deposits were carefully examined.

But in order to leave no vestige of doubt on this point, Mr. Everitt contrived a little apparatus, by means of which the vapours emitted by a suspected candle in a state of ignition, were obtained, partly in a solid form, adhering to the inside of the body of a retort; and partly dissolved in the condensed steam deposited in the horizontal tube of the same retort, which was kept, for that purpose, in a constant state of refrigeration. Under both those forms arsenious acid was detected.

It is to the interesting question of what are the productions of the combustion of arsenicated animal fats, that one of the members of your Committee, Mr. Golding Bird, chiefly directed his attention. He first experimented on arseniferous gases, and afterwards instituted some direct trials with a mass of fat, in which arsenious acid was mixed, and which by means of a wick was set on fire. In watching the operation in both cases, Mr. Bird convinced himself of the fact, that according as the combustion is impeded or free—that is, according as more or less oxygen has access to the flame—metallic arsenic—or the so-called black oxyde of arsenic (?)—or arsenious acid, is given out and deposited under their respective characteristic forms. According to Mr. Golding Bird's experiments, there might be a point of such low combustion in the burning of arsenicated fats, as to give rise to that most deleterious and fatal gas called arsenuretted hydrogen gas.

In the course of their analytical enquiries, your Committee received specimens of candles for examination, from clubs, institutions and private families, some of which were found to be arsenical, while others were not so. And in order not to leave any point undetermined, the analysis, in some instances, was extended to wax, spermaceti, and the old-fashioned composition candles, in none of which the noxious material was detected.

It would be superfluous to specify more minutely or technically to the Society, the several operations gone through by your Committee, with a view to settle the chemical question of the presence of arsenic in the above candles. It is sufficient to state, that the fact of its presence in such candles was established beyond all possible doubt, and that the quantity contained is considerable.

Your Committee next directed their attention to the best mode of ascertaining, as far as such an investigation can admit of demonstration, the probable effect which the respiring of the ascertained quantity of arsenical vapour might have on animal life; and after some consideration, it was determined to expose various living animals to an atmosphere in which arsenicated, or Stearine candles, were burning, at the same time that an equal number of the same species of animals, and, as nearly as possible, of the same age and strength as the first set, were placed in an atmosphere of similar dimensions, wherein spermaceti candles only were used."

Here follow the experiments, which were varied and satisfactory, being conducted with the greatest care and precaution.

"With respect to these seven birds, which died in the course of a week, your Committee will only offer a few general observations, in addition to the symptoms already specified. First, it was remarked that they drank at least four times as much water as the other birds, not exposed to the arsenicated candles; that when they had taken a seed into the beak, and broken the shell, they resorted to the water, and immersed the bill before they swallowed the seed; that they gradually lost their inclination for food; and, lastly, that they were affected, during the best part of the experiments, with diarrhœa, accompanied by a continual propulsive and retractive action of the anus. The discharge was a greenish serous fluid, very different from the fæculent matter of birds.

It has been stated, that during the combustion of arsenic associated to animal fats, in which both hydrogen and oxygen are present, the three possible states in which vapours may arise, according to the degree of combustion going on, are: first, arsenuretted hydrogen gas; secondly, a vapour holding a mixture of metallic arsenic and its so called black oxide; and, lastly, a vapour of white oxide of arsenic or arsenious acid. The first is only a possible, but not a very probable occurrence. When it takes place, its effect on animal life is quickly fatal. The second can hardly take place at the temperature at which the combustion of Stearine candles goes on. But the third is a more general case, and it is to its effect on health and life, that the society need direct their attention.

What may be the condition of the atmosphere around a vessel or a burning candle, emitting vapours holding black oxide of arsenic, as it has been called, your Committee are not able to state, nor is it to their purpose to enquire. Still they find it asserted by Dr. Merat, in his voluminous work on Materia Medica, that the *poudre à mouches* (which is the preparation of arsenic alluded to), employed in France to destroy flies, is fatal to them, if they but approach the atmosphere around the vessel containing its solution."

Then follow several historical instances of the deleterious effects of the vapours of arsenious acid on the human frame, which we are unable to quote, but which cannot possibly excite a doubt in the mind.

"Such being the case, is it too much to assume, that whenever a large number of candles of arsenicated Stearine are lighted in a room, a club-house, an assembly, a theatre, or a church, filled with people, who remain for some hours exposed to the vapours arising from the combustion of those candles, mischief to the health of some, at least, of the parties, may be expected? Let us suppose that the interior of Drury-Lane Theatre, whose brilliant lustres hold exactly one hundred and fifty-two candles, were to be lighted with Stearine candles for cheapness' sake, and under the impression that such candles are better looking than other composition candles, burn better, last longer, and give a clearer light; (all which has been promised by their manufacturers, but which your Committee have found from experience not to be quite correct;) in that case 608 grains of arsenious acid would be vaporized, and float in the air during the time of the performance. Is any one prepared to assert, that not one of the individuals present on such occasions would receive the slightest injury from an arrangement of this kind, and from the subtle particles of the arsenic wafted to and fro, through the atmosphere of the house, by the system of ventilation employed in it?"

Numerous documents are then brought forward by Dr. Granville, shewing the attention of the French government to this subject, which government took care to stem the evil of arsenical candles in its embryo by legislative measures.

"In spite of the length to which their Report has already extended, your Committee deem it essential to the completion of their inquiry, to conclude with some useful and practical remarks.

The various specimens of candle examined by your Committee were either procured at different shops, or were given to them under the following several appellations:—Stearine candles—German wax—Imperial wax candles—French candles—Pressed tallow—tropical candles—moulded wax—and Venetian wax. The Committee have been told (but of this they have no personal knowledge) that candles of the same description were sold, some time ago, under other denominations, such as adamantine candles, pearl candles, &c. In fact, each candlemaker thinks it essential to give a different name, as well as to affix a different price to his own candles made of one and the same material; namely, Stearine. This substance, when in its saponified and crystallized form, being brittle, arsenic is added to harden it, as well as give it more adhesion. The Committee had two Stearine candles made without arsenic, and certainly the difference in appearance and feel of the candle, was in favour of those which contained arsenic. The effect, however, sought to be produced by arsenic, your Committee are led to believe, may be produced also by a very small proportion of wax, as in the case of spermaceti candles, which cannot be made for the same reason, without using a thirtieth part at least of wax with it—for *Cetine*, the principle of spermaceti, likewise discovered by Chevreul, possesses, equally with pure Stearine, the particular brittle grain complained of.

Your Committee have learned from an experienced manufacturer, that for ten dozen of pounds of candles of Stearine, one pound of arsenic is employed. In order to make it intimately mix with Stearine, and produce the desired effect, the temperature of the latter must be kept at 200 deg. of Fahrenheit. The wick, also, which is platted, is dipped in diluted sulphuric acid, till it is nearly rotted,—and a little gamboge is added to the mass to colour it yellow in imitation of wax. This imitation is farther strengthened by the opacity, which arsenic seems to impart to Stearic acid. But the public need not be deceived in the purchase of such candles—first, because the lowness of their price is in itself sufficient to show that real wax, or anything approaching to it, cannot form the least part of such a candle,—and secondly, because there are characteristic features in the Stearine arsenicated candles which happily distinguish them from all others. With regard to the price, however, that is not always a sufficient guarantee, for it has come to the knowledge of your Committee, that one of its members, upon sending for a genuine wax candle at a shop, got one as like to wax in appearance, as it was in price, but which proved to be an arsenicated Stearine candle! The latter may in a moment be distinguished from wax by the platted wick, which has never been yet used in wax candles; and from spermaceti candles, first by the transparency of the latter, and secondly, by this general character, that when the surface of either a spermaceti or a wax candle is rubbed backwards and forwards three or four times, with the edge of an ivory knife, the polish or lustre is much heightened; whereas the surface of the Stearine candle, treated in a similar manner, loses the slight polish it naturally has, becomes dull, and no effort can restore the lustre equal to that of the other parts of the candle.

The fractured surface too of the Stearine candle suddenly snapped in two, presents a very different aspect from that of the fractured surface of either spermaceti or wax candles. The latter, or the wax candle, exhibits regular concentric circles, or circular laminæ around the wick; the other, or the spermaceti candle, looks like a broken piece of camphor or a broken watery turnip; whereas the fractured surface of the Stearine arsenicated candles looks spungy, is easily rubbed into a white powder by the finger-nail, and, seen through a magnifying-glass, presents minute shining particles.

The presence of the arsenious acid, however, for immediate and practical purposes, may be detected, first by the garlic-like smell, which is perceivable when the candle is extinguished, while part of the wick is red-hot; and secondly, by placing, for a quarter of an hour or twenty minutes, over a suspected candle, (the flame of which should be all the time perfectly steady,) a small bell-glass or shade, on the inside of which, if arsenic be present in the candle, a white powdery deposit will take place. This last experiment, however, is not of easy execution.

With respect to the *alliaceous* smell, every writer and experimentalist seems to have agreed upon its being an excellent popular test for warning people that arsenic is present. Your Committee, therefore, recommend, that it should be assumed, where it exists in candles, as a sufficient reason for at once discarding them. There are those who imagine, that if zinc were used in the Stearine candle, instead of arsenic, the smell, on extinguishing them, would be the same. In order to settle this question, your Committee, on two different occasions, made some direct experiments, by burning a wick in Stearine, in which white oxide of zinc had been abundantly mixed; but they could not perceive the smallest trace of the peculiar alliaceous, or garlic-like odour in question.

Dated the 8th of December, 1837.

Signed,

J. JOHNSON, M.D.
A. T. THOMSON, M.D.
CHARLES J. B. WILLIAMS, M.D. F.R.S.
WM. B. COSTELLO, M.D.
JAMES SCOTT, M.D.
R. PHILLIPS, F.R.S. &c.
GOLDING BIRD, F.L.S.
THOMAS EVERITT,
A. B. GRANVILLE, M.D. F.R.S. &c. *(Reporter)*."

In an Appendix the minutes of the different sittings of the Committee are regularly reported.

---

APPENDIX TO THE EIGHTH EDITION OF THE PHARMACOLOGIA, WITH SOME REMARKS ON VARIOUS CRITICISMS UPON THE LONDON PHARMACOPŒIA OF 1836. By *J. A. Paris*, M.D. Highley, 1838.

THIS Appendix consists of three parts—the *first*, a vindication of the College against its Pharmacopœial critics—the *second*, a double list of the old and new names in the recent Pharmacopœia, with a table of new medicines—and *third* miscellaneous addenda, or remarks on a number of articles and compounds in the Pharmacopœia and Pharmacologia.

In the first part the College has found an able apologist, or rather advocate. That BODY has many enemies—some, perhaps, of its own making—and others who are not open and candid opponents of what they consider grievances or defects, but enemies who lie in wait, with the daily, or at least, annual prayer in their mouths,—"Oh that my enemy would write a book!" The College does not very frequently gratify these snarling critics; but when it does, there is instantly a hornet's nest broke open, and the stinging trade flourishes for six or more months, with wonderful vigour. Among the College censors (not the censors of the College) there are some who might well be called, "nimis acer et ultra;" who, not content with carping at certain preparations and directions in the Pharmacopœia, would annihilate it *in toto*, as a drag on the march of intellect. To these destructives the following arguments are pertinent—and, indeed, they are unanswerable.

"As to many of the Pharmacopœia reviewers, when we perceive the captious style, the uncalled-for censure, the scorn, the sneer, the reluctant admission of improvement, and the pertinacious adherence to refuted error,—every intelligent and candid reader will justly appreciate the spirit and motives of such critics. In one instance, the attack is opened with vituperations upon the order in council prefixed to the work, by which 'His Majesty doth strictly require, charge, and command, all apothecaries and others' to obey its direction, &c. Why, in the name of common sense, let me ask, for what purpose is the Pharmacopœia issued, unless an obedience to its orders can be enforced? Uniformity in the composition and strength of our medicines are its object and purpose; but, says the critic, 'it is a *drag-chain* upon science; every hour ushers in new improvements, which are applicable to all processes;' and he adds, 'instead of being encouraged to make discoveries, the English chemist is tied to the prosecution of stale processes, which he is not allowed to improve:' and truly fortunate is it for the community, that such a check, such a patent '*drag-chain*,' has been mercifully provided for its safety. What would be the consequence, were every manufacturer, who fancied that he had invented a superior formula, allowed to adopt it? —to what a wild and dangerous spirit of speculation and empiricism should we be thus consigned! The physician could never calculate upon the effects of his remedies; for it is probable that no two laboratories would furnish the same article. Even in the page of the critic who advocates so preposterous a doctrine, we may discern traces of self-conceit, that would lead him, unless restrained by our *safety-drag*, to give a dangerous activity to the *arsenical solution*, by what he is pleased to consider an improved formula for its preparation. In many of those processes, however, which are more immediately the objects of the wholesale manufacturer, and where the article is readily tested as to purity and strength, he is not tied down by the Pharmacopœia to an invariable form of preparation, provided always that the product be what is required; and hence the introduction of a series of 'notes,' by which its purity may, as nearly as possible, be ascertained; but this measure of precaution did not supersede the necessity, or render less useful the introduction of formulæ, in a work expressly composed for the instruction and guidance of the pharmaceutic chemist. But let us view the question in another point of view. In a system founded upon a division of labour, there is an implied condition between the different departments, that each shall fairly and honestly work with the other, for one common purpose, and to one common end. In the case before us, that purpose and that end is the welfare of the community. The medical men by whom the public are served, may be divided into two distinct classes,—those who prescribe remedies, and those who prepare them. In the former class may be included physicians and general practitioners; in the latter, manufacturing and dispensing chemists. Now let us inquire how these respective classes stand, in relation to the Pharmacopœia,—ever keeping in mind, that they are to act in union for the benefit of the public. As this question has been raised by our reviewer it is necessary that a few remarks be made upon it. He asks, 'for whose use is the Parmacopœia published,—is it for the use of the learned physician?' he answers, 'surely not; his accomplishments and opportunities of acquiring knowledge place him above such a source. Is it published for the benefit of the operating surgeon? No,—the lancet and the scalpel are his medicaments; and when he has to call in the assistance of pharmacy, his scientific resources place him in a condition similar to that of the physician: for whose use, then,' he inquires, 'is the Pharmacopœia destined, since it is neither required by the physician nor by the surgeon?—the truth is,' says he, 'it is published for the use of the general practitioner, and the chemist, and druggist;' neither of whom can, in the reviewer's judgment, 'be expected to waste his leisure in studying a Latin Pharmacopœia.' All this appears to me perfect trash and nonsense. The general practitioner, unless he possess sufficient knowledge to understand the Pharmacopœia, is a person unworthy of being trusted by her Majesty's liege subjects; and as to the druggist or chemist, whose sole business it is to dispense prescriptions, it is surely not too much to expect that he should be able to read them. But how does the question really stand? Certainly not as our critic represents it. The Pharmacopœia is obviously constructed for the physician, or prescribing practitioner; his knowledge, however 'extensive and transcendent,' does not render such a recognized standard the less necessary or convenient. It abridges his labour, without cramping his resources; for he is still at full liberty, should it so please him, to range over the whole creation in search of remedies. He is, however, here presented with a list of the medicines which, under all ordinary circumstances, he can be supposed to require, and which he may combine and modify at his will and pleasure; while the dispenser, by being thus made acquainted with the simple and compound articles which in the daily routine of his business he will be called upon to furnish, is better able, by promptitude as well as accuracy, to fulfil the intentions of his master. What a sacrifice of time, and what multiplied chances of error, does this system of mutual accommodation obviate! The critic who stands forward to arraign its expediency, may, for ought I know, possess all the learning so cynically assigned to the physician, but he can be no practitioner; and it may be shrewdly suspected, that he is better qualified to publish sarcasms than to write prescriptions."

To the truth and reasonableness of the foregoing observations, we think every candid mind must subscribe. Order, even with many imperfections, is preferable to anarchy; and, if we had no standard Pharmacopœia, to which the chemist is bound to adhere, there would be no security for prescribers—no, not even for the general practitioner, who dispenses, but does not prepare his own medicines.

Many object to the Pharmacopœia being written in Latin. But while physicians prescribe in Latin, the Pharmacopœia ought to be written in that language. We hope it will always be in a dead language. There is already too much prying curiosity on the part of the patient, as to the names and properties of the ingredients. And the more he knows of these, the more doubt and distrust he has in the prescription, and the less efficacious it is likely to prove. Every one who knows human nature is aware of the fact—" Omne ignotum pro magnifico"—and no man has had much practice, especially among the better classes of society, who has not seen the most efficacious and appropriate remedies rendered a nullity by the prejudice of the patient, and the prejudice generally founded on some superficial knowledge of the medicine prescribed. On this account the general practitioner has a great advantage over the physician and surgeon; and it would be still greater, if the present system of making the whole charge to rest on the drugs, was ameliorated. On the Continent, where the prescriptions are generally (especially in France) written in the vernacular language, there is not, comparatively speaking, a proper confidence in the medicine; and too much importance is attached to diet and regimen.

Independently of this view of the case, we hope that Latin and Greek, but particularly the former, may always be considered indispensable in the medical student's education.

"I shall, therefore, only express, more in sorrow than in anger, a deep regret, that any writer who has at heart the interests and respectability of his profession, should seize upon every occasion to pander to the vulgar taste of obliterating whatever has the semblance of learning. It is the character of the mischievous goose, *improbus anser*, to tear up by the root every thing it approaches, '*et morsu lædit et stercore*.'"

The nomenclature, or change of names of medicines in each edition of the Pharmacopœia, is more debateable ground. It certainly is attended with great inconvenience, and, we have no doubt, with many disastrous consequences, for some time after each issue of the work. Those practitioners who are above forty or fifty years, are unwilling to learn new names for old remedies, and change their language of prescription. Thus, when the term CALOMEL was thrown out, and Submurias Hydrargyri substituted, the College only changed one bad name for another, since the new name was subsequently changed for Hydrargyri Chloridum—and who can say that this will hold its ground many years. But this is not the worst. Between calomel and corrosive sublimate no mistakes could possibly happen from any similarity of names; but the Submurias and Oxymurias were often confounded, and fatal results occasionally happened. The resemblance in nomenclature is now still more close—H. Chloridum and Bichloridum.

But it may be fairly urged by the censors, that because chemical science is *progressive*, nomenclature should not stand still. Every change in the *latter* indicates a step in advance of the *former*. And if old men will not go to school again, they can jog on with old names during their day, while the young are sure to adopt the new nomenclature.

The third part gives us a complete list of the new names, and the old ones changed. We think the *double* list was hardly necessary—namely, first the new and old, and then the old and new. This plan, however, facilitates reference.

The addenda contain various notes and remarks, rendered necessary by the lapse of time since the last edition of the Pharmacologia was printed. The following specimen of these addenda will be sufficient in this place :—

"CREOSOTON.

"When pure, it exists as a limpid colourless liquid, having a sp. gr. of 1·037. It has a hot pungent taste, and a peculiar empyreumatic odour. It boils at 397°, and does not congeal at—50°. It is soluble in 80 parts of water, and in every proportion of alchohol, æther, and acetic acid. It appears to be a compound of oxygen, hydrogen, and carbon, in unknown proportions, and hence is described, in the Materia Medica, as an *Oxy-Hydro-Carburet.* It derives its name from its property of preserving animal matter; and the antiseptic effect of smoke-drying hams, and other articles, depends, probably, upon its agency. By the believers in the virtues of Tar-water, if such there be, this article will be received as an elegant and refined concentration of that remedy. It has been happily observed, that science progresses in a spiral; for although, in our revolutions, we may appear to be returning to the point from which we started, we are in truth always advancing. Creosote has been stated to possess singular powers in arresting vomiting; but I must confess, that in all the trials I have made with it, I have met with nothing but disappointment. Like an essential oil, it is stimulant, and may therefore possibly prove useful in certain gastric affections. Dose, m. v—xv. Externally, it has been applied in cases of Porrigo, and other cutaneous affections, *(Unguentum Creosoti)*, and also as a lotion to foul and indolent ulcers."

To those who hold the last Edition of the Pharmacologia, (and they must be numerous,) this Appendix will be welcome, as it may easily be bound up with the volume. It is not undeserving of attention also from the critics who have so vehemently assailed the Editors of the Pharmacopœia.

---

A Practical Treatise on the Curative Effects of Simple and Medicated Vapour applied Locally, &c. &c. By *James Wilson*, M.D. Churchill, 1838.

The Author of this unpretending little work was a pupil of Dr. Macartney, of Dublin, many years ago, and freely acknowledges that his attention was directed to the above subject by the chemical lectures and precepts of his quondam master. The Author has since put these precepts into practice, and now communicates the results to the public.

The apparatus used by our author are sufficiently simple—consisting of a vessel in which the vapour is generated—a tube to convey it—and a suitable contrivance adapted to receive it and retain it round the affected part. This apparatus Dr. Wilson offers to shew freely to any of his professional brethren, in Sackville-street.

"The only long standing and well known instances of the local application of steam, are fomentation and poultice. Even of these, it is only a few who consider their action in a true light,—which is, a medium for the application of warmth and moisture, or vapour, to the affected part. Objections, however, arise to these excellent remedies, when compared with the immediate application of steam unmixed with solid matter. In the first place, the weight of the foment-ing flannel, or of the poultice, is objectionable, particularly when an abraided, ulcerated, or exceedingly sensitive part is involved. I have seen a white-swelling of the knee, and a cancerous ulcer, in such an exquisite state of sensibility as to render the weight of a poultice nearly intolerable. In the next place, it is impossible to make the application of either of these means *uniform and continuous*. The flannels grow cold, and then are changed for hotter; thus marring the very end proposed, which is, to act gently, soothingly, and therefore with an *uniform* agent, on the sensibility of the part. A poultice is in the same predicament, never retaining the same degree of heat for a long time together; its moisture, too, varies,—for on an inflamed surface it rapidly becomes dry, and a source of pain. In the third place, the difficulty of applying medicinal substances in fomentation and poultice is considerable, as they are apt to act too violently, or to be altogether useless, according to the temperature of the water or poultice. Again, composed, as a poultice usually is, of some farinaceous substance, it is liable to undergo chemical changes, and in addition to its commixture with the secretions of the part to which it is applied,—an ulcer, for instance,—to become sour, and thus to prove irritating where soothing is required. That such is the fact, the experience of any surgeon must witness. For myself, in former years, I could never account for the slow, unsatisfactory operation of a remedy the principle of which was good, until upon reflection the advantages above recited rose in strange contrast with the advantages set forward by Professor Macartney, of the same principle in a different mode and form. That form was what Dr. Macartney called his water dressing, and watery vapour, of various degrees of temperature,—the beneficial, and, I may add, extraordinary effect of which I have now verified in such a considerable number of cases as to induce me to reject the employment of fomentations and poultices, and substitute the above in all instances where it is practicable.

"In order to make it so in the great majority of local disorders, I have contrived variously shaped envelopes, composed of waterproof substance, which being applied to the head, the shoulder, the breasts, knees, feet, genitals, or any other affected part, receive vapour of higher or lower temperature, and enclose it continuously over the surface. Of the facility of the application of this remedy, and its wonderfully sedative influence on the morbid sensibility of a part, none can accurately judge but those who have beheld it in operation. I have seen inveterate rheumatism of the knees allayed, as if by magic, by four or five steamings, —the skin of the joint appearing after each application as if it had been soaked in hot water for days, whereas the application had continued for an hour and a half only. In long-standing ulcers, particularly of a painful character, it both changes the morbid secretions, and diminishes the excessive sensibility."

The temperature of the vapour must be regulated according to the common and organic sensibility of the part. Dr. W. has contrivances for impregnating the vapour with medicinal substances. The following catalogue is presented by the Author, according to the scale of sensibility.

"1. Local diseases in which the sensibility of the part is morbidly exalted:

Erysipelas.
Piles.
Branny Scall (Porrigo furfurans.)
Lupine Scall (P. lupinosa.)
Honeycomb Scall (P. favosa.)
Scalp Ringworm (P. scutulata.)
Irritable and sloughing Ulcer.
Cancerous Ulcer.
Acute gouty inflammation of the feet and hands.
Whitlow.

2. Local diseases in which the sensibility of the part is deficient:

   Bald scalled head, (Porrigo decalvans.)

   Indolent Ulcer.

3. Local diseases in which the sensibility of the part is deficient externally, in consequence of its augmentation internally:
   White-swelling of the knee and elbow.
   Chronic synovial inflammation of the knee.
   Chronic rheumatism of the knee, elbow, shoulder, and ankles.
   Inflammation of the prostrate gland and neck of the bladder.
   Gonorrhœa."

On the foregoing list Dr. W. comments seriatim, and lays down practical rules for the application of vapour in each individual case. For all these we must refer to the volume itself, which is sensibly written and completely free from the slightest tincture of charlatannerie.

---

Elements of Anatomy. By *Jones Quain*, M. D. Fourth Edition, Revised and Enlarged. Illustrated with Steel Plates, and numerous Engravings on Wood. Parts I. II. and III. Price 5s. each.

Dr. Quain's Work on Descriptive Anatomy has been for some years before the Profession. Its success has been considerable, yet not greater than it merits. We have, on former occasions, pointed out the principal features that distinguish it, and the slight defects that appear in it. It presents a good epitome of the existing state of anatomical knowledge both on the Continent and in this Country, and Dr. Quain judiciously improves each succeeding edition, by transferring to it all that has been done in the way of anatomical discovery since the publication of the preceding one.

The present edition is particularly characterized by the wood-cut illustrations that appear upon its pages. Their number is not inconsiderable. In 698 pages of letter-press, we find 150 wood-cuts. There can be no question of the great assistance which they give to students, particularly to that poorer portion who are not enabled to purchase systems of anatomical plates.

The style of Dr. Quain is simple, clear, concise; and he generally avoids that indescribable sort of composition which prevails so much in our medical cyclopædiæ and elementary works, and makes us doubt while we peruse it, whether it is really intended for English. He has had the good sense to abbreviate the details of unimportant particulars, which we find swollen to so unconscionable a length in the works of Cloquet, and especially of Bourgery and Cruveilhier.

On the whole, these Elements of Anatomy are eminently calculated to be popular with students, and, indeed, they are so.

---

Elements of Physiology. By *J. Müller*, M. D., Professor of Anatomy and Physiology in the University of Berlin, &c. Translated from the German, with Notes, by *William Baly*, M. D., &c. Illustrated with Steel Plates, and numerous Wood Engravings. Part II., containing Secretion, Digestion, Functions of the Glands without Efferent Ducts, and Excretion. Taylor and Walton, London. Price 3s. 6d.

In a Notice to this Part, we are informed, that, " since the publication of Part I. of this Translation, a new edition of that portion of the original has appeared in Germany, the most important changes in which relate to the subject of Respiration. In order to give the purchasers of the present Work the advantage to be derived from these changes, the pages from 321 to 334 have been re-translated and introduced at the end of the present Number. The same pages in Part I. should therefore be cancelled when the book is bound, and these substituted."

This, at all events, proves the anxiety of the Translator to do all the justice in his power, both to the author and the English reader.

For reasons which we need not state at present, we shall merely recommend this work most strongly to our readers. We shall not attempt, in *this* Number of our Journal, to present any account of it. We *do*, however, recommend it very strongly, and we have no hesitation in saying that it will supersede all other works as a text-book for lecturers, and one of reference for students. It steers a middle course between the superficial brevity of many of our own works, and the pleonastic and metaphysical jargon of Burdach.

Part III. will be published in the course of May. The price of the work is guaranteed not to exceed £1. 7s.

---

MEDICINE AND SURGERY ONE INDUCTIVE SCIENCE; BEING AN ATTEMPT TO IMPROVE ITS STUDY AND PRACTICE, ON A PLAN IN CLOSER ALLIANCE WITH INDUCTIVE PHILOSOPHY, AND OFFERING, AS FIRST FRUITS, THE LAW OF INFLAMMATION; ADDRESSED PARTICULARLY TO THE MEDICAL STUDENT AND THE PROFESSION, BUT EASY AND INTELLIGIBLE TO THE PUBLIC ALSO: THE WHOLE BEING THE INTRODUCTION AND FIRST PART OF A SYSTEM OF SURGERY. By *George Macilwain*, Fellow of the Royal Medical and Chirurgical Societies, Surgeon to the Finsbury Dispensary, Consulting Surgeon to the St. Ann's Society, &c. London, S. Highley. 1838.

THIS Work consists of 551 closely printed pages. The author informs us that it is the " Introduction and First Part of a System of Surgery." We fear that that System will be voluminous, too voluminous indeed for the mass of superficial readers and thinkers of this day. Yet Mr. Macilwain has not laboured to amplify, but to condense his book.

" The size of the volume," he says, " as in manuscript, has been considerably reduced,—1st, by the omission of authorities, which would have increased its size and expense, and have made a parade of learning without adding to its utility; 2ndly, by reducing the description of the body, originally intended to have been given, to a very few general remarks, for which the object must be the apology; 3dly, I have not scrupled to omit, in aid of the reduction in size, many arguments and illustrations in support of the law of inflammation. Nothing would have induced me to do this but the following considerations:—I am much more desirous of exciting reflection, than of at once carrying conviction; and the facts and arguments omitted will, from time to time, appear in connexion with different diseases. I am not in the least degree anxious that my views should be hastily adopted: nothing is more injurious than the reception of propositions without examination."

Mr. Macilwain, it will be noticed from the title-page, addresses his book to three distinct classes of readers—the profession—those who are entering it—and the public. He laments the difficulty of making what he writes both intelligible and adapted for all. We agree with him in thinking this impossible.

The merits and the defects of the work lie in its generalizations. They are very broad. We will not pretend to have examined them closely. It would not be fair to Mr. Macilwain to discuss them, unless we discussed them fully. It would not be fair to our readers to do that. Without, then, meaning any disrespect to Mr. Macilwain, we fear that his work will prove too voluminous and too general either for reviewers or for any large class of readers. We commend it to the philosophical few, possessed of leisure and of inclination for the examination of the inductive reasoning which its author offers. His object is a good one—to render medical reasoning more exact.

The Philosophy of the Eye; being a Familiar Exposition of its Mechanism, and of the Phenomena of Vision, with a View to the Evidence of Design. By *John Walker*, Author of "The Principles of Ophthalmic Surgery," Lecturer on the Eye in the Manchester Royal School of Anatomy and Medicine, and one of the Surgical Officers of the Manchester Eye Institution. &c., with numerous Illustrations. London, Charles Knight. 1838.

The object of this work is briefly but sufficiently explained by its Author in his Preface.

"Various successful attempts," he says, "have been made, during the last few years, to popularize a knowledge of the structure and functions of the animal body, particularly by Sir Charles Bell, Dr. Roget, and Dr. Southwood Smith. Among the many portions of the subject that have attracted the notice of these eminent men, that of all others the most interesting—the Eye—can scarcely be regarded as having met with the degree of attention to which its admitted claims would seem to have entitled it. Hitherto there has been no separate treatise to which the general reader could refer for an ample and satisfactory account of this organ; although surely, if there be any one in particular that deserves to be isolated and considered apart from the other animal organs, it is that which contributes to vision, the noblest of the senses.

It is the special object of the present undertaking, then, to supply the presumed want of a separate and familiar treatise on the mechanism and functions of the visual apparatus, including the Eye and its appendages.

The Author has felt it impossible to perform the task which he had set himself without frequent references to the evidence of design, so abundantly furnished by the Eye. However, he has scarcely allowed himself to do more than incidentally touch upon this most interesting topic,—a topic which has been so ably discussed, and indeed almost exhausted, by the learned and accomplished writers of the Bridgewater and other similar Treatises."

The descriptions are sufficiently illustrated by wood-cuts, and the work is altogether well got up, and well adapted to serve the useful objects of the Author. Being addressed to the Public, it is not adapted for more particular notice.

---

A Treatise on Ruptures. By *William Lawrence*, F. R. S., Surgeon Extraordinary to the Queen, &c. &c. &c. 8vo. The Fifth Edition. Pp. 632. Churchill, 1838.

The present edition has been much enlarged, and, we understand, in a great measure re-written. It is, we think, a pity, that, in issuing new editions, authors do not make a point of stating what *is* new. It is hard on a reviewer to be compelled to compare two bulky volumes, in order that he may distinguish what is altered or added.

The work of Mr. Lawrence is so justly deemed a standard one, that it would be idle to dwell upon its merits. All that we need do is to announce this new edition, and to recommend those who have no other to purchase it.

---

Elements of Chemistry, including the recent Discoveries and Doctrines of the Science. By the late *Edward Turner*, M. D. Sixth Edition, enlarged and revised by Justus Liebig, Professor of Chemistry in the University of Giessen, and William G. Turner. Part II. Taylor and Walton, 1837.

This Part is devoted to the Metals, and embraces Crystallization, and the consideration of the class of Salts.

We noticed the first part of this, decidedly the best work for a student, on its first appearance, and we are glad to announce the appearance of the second. There has been so much delay in the publication of "Parts" of works, that purchasers have naturally become suspicious.

We cordially recommend our readers to possess themselves of this excellent manual of chemistry, and we think they may lay out their money on the Parts already published, in the full assurance that the future ones will appear in reasonable time.

---

### RECENT WORKS ON THE PRACTICE OF SURGERY.

I. INSTITUTES OF SURGERY: ARRANGED IN THE ORDER OF THE LECTURES DELIVERED IN THE UNIVERSITY OF EDINBURGH. By *Sir Charles Bell*, F.R.SS. L. & E.; Professor of Surgery in the University of Edinburgh; late Professor of Anatomy and Surgery to the College of Surgeons of London; and Surgeon of the Middlesex Hospital; Consulting Surgeon of the Royal Infirmary of Edinburgh; Honorary M.D. of Gottingen, &c. &c. In 2 vols. *Edinburgh*, A. & C. Black; *London*, Longman, & Co.

II. PRACTICAL SURGERY. With One Hundred and Twenty Engravings on Wood. By *Robert Liston*, Surgeon. *London*, John Churchill; H. Renshaw.

HERE are works by two Surgeons of eminence and talent in their respective ways. It would be an injustice to both to limit our account of their works to so brief a notice as this: nor shall we do so. We merely introduce the works to our readers at present; in our next number, we shall offer a more full account of them.

The preface of Sir Charles Bell deserves to be read, we may say studied, at this day. In a few sentences, it attacks three vices of the times—the affectation of despising all ordinary routes to knowledge—the practice of denouncing the mistakes of others, as if we really made none ourselves—and the writing *for* practice, not *from* it. Let Sir Charles Bell speak, and our readers listen.

"The study of Surgery, an art unexampled in the weight of responsibility which it imposes, must be prosecuted in more than one way,—at lectures, by attendance on the hospital, and by reading. There must be a book of ready reference. That book to be useful must recall to the reader's recollection the demonstrations and the reasonings which he has heard at lecture; for no book can be written which is sufficient for the practice of the profession: And it should state correctly, however shortly, the rules which are to guide the judgment and the hand of the practitioner, in the moment in which he is called upon to the performance of his high duties.

The book should also contain references to some of the best authors, with a recommendation of those which ought to be read. But it is, above all, important that such a work should contain so much of criticism at least, as may guard the reader against the bad influence of writers who have not known the hazards and difficulties of practice; who make unhesitating and bold assertions, where good and experienced men hesitate and are afraid; who write rather to obtain a name (an object not altogether to be condemned) than to guard their successors against the errors which they have witnessed, or to detail the means by which they themselves have happily succeeded."

The Preface of Mr. Liston is deprecatory, probably to an unnecessary extent, and pleasant. It corrects no vices, nor does it heed them. He insists much upon the wood-cuts, a mode of illustration which has grown fashionable, and, when judiciously employed, is useful.

